# Corrosion Behavior and Deterioration Mechanism of Ecological Concrete in Deep Water Environment

**DOI:** 10.3390/ma19112255

**Published:** 2026-05-26

**Authors:** Hua Wei, Kang Wang, Chunhe Li, Anyi Chen, Hao Lu

**Affiliations:** 1 Materials & Structural Engineering Department, Nanjing Hydraulic Research Institutes, Nanjing 210029, China; hwei@nhri.cn (H.W.);; 2 Department of Civil and Environment Engineering, The University of Miyazaki, 1-1 Gakuenkibanadainishi, Miyazaki 889-2192, Japan; lichunhe@cc.miyazaki-u.ac.jp

**Keywords:** ecological concrete, pressure-driven penetration, chloride ion migration, composite corrosion, deterioration mechanism

## Abstract

To elucidate the corrosion behavior of cracked reinforced concrete under pressurized permeation at 0.3 MPa, three exposure conditions were established, namely a low-concentration chloride-sulfate solution, a high-concentration chloride-magnesium sulfate solution, and a high-concentration chloride-magnesium sulfate-carbonate composite solution, and cyclic permeation tests were conducted. Based on the chloride ion (Cl^−^) content distribution, permeation behavior, and steel half-cell potential measurements, together with XRD and SEM microstructural analyses, the results showed that Cl^−^ transport was significantly enhanced. At a depth of 15 mm, the Cl^−^ content increased from 0.011% under unpressurized conditions to 0.211% at 90 d and further to 0.322% at 365 d, while it remained close to zero at depths of 35 mm and below. Under the low-concentration condition, permeation ceased after about 3 months, and the half-cell potential recovered from −550 to −580 at 46 d and stabilized at −250 to −340 in the later stage, indicating a certain self-healing capacity of the cracks. Under the high-concentration and composite conditions, permeation persisted throughout the 12-month period, and the half-cell potential dropped to −800 to −900 at 34–40 d and remained at −580 to −840 in the later stage, indicating that the steel remained in an active corrosion state for a long time. XRD and SEM results further showed that corrosion products such as AFt increased, and the internal concrete structure gradually deteriorated.

## 1. Introduction

Reinforced concrete, with its high load-bearing capacity, good integrity, and strong engineering adaptability, is widely used in infrastructure such as bridges, underground engineering, ports, and marine engineering. However, traditional reinforced concrete still faces problems such as high resource consumption, high carbon emission from cement production, and susceptibility to environmental effects during long-term service [1,2,3]. Especially in underground engineering and marine engineering, structures are often in complex water environments containing multiple corrosive media such as chloride salts, sulfates, and carbonates. Once cracks occur in concrete, external corrosive media can more easily penetrate into the interior along the cracks and pores, thereby accelerating rebar corrosion and material deterioration, and significantly shortening the service life of the structure [4,5,6,7,8]. This special concrete material, which emphasizes harmonious coexistence with the ecological environment throughout its entire life cycle, has environmental protection functions such as resource conservation and utilization of solid waste, as well as ecological functions like ecological restoration and environmental improvement [9,10].

In recent years, to reduce the environmental load of traditional concrete and improve the resource utilization of industrial solid waste, a large number of studies have been carried out on ecological concrete with industrial solid wastes such as fly ash, slag powder, and steel slag as the main mineral admixtures [11,12,13,14]. Existing studies have shown that the addition of mineral admixtures helps to reduce the amount of cement clinker and carbon dioxide emissions, and at the same time can improve the paste microstructure through pozzolanic reaction and potential hydraulic reaction, thus improving the compactness, late-age strength, and impermeability of concrete [15]. Therefore, ecological concrete has a good application prospect in the field of green and low-carbon building materials and high-performance durability materials.

Nevertheless, the durability of ecological concrete is still significantly affected by the service environment and transport conditions. Different from general atmospheric exposure or non-pressure immersion conditions, in deep water environments, underground confined water environments, and marine environments affected by seepage, concrete is often subjected to external water pressure and multi-ion composite erosion at the same time. The water pressure will significantly enhance the migration capacity of corrosive media in cracks and pores, making corrosive components such as Cl^−^, SO_4_^2−^, Mg^2+^, and HCO_3_^−^ enter the concrete interior faster, and more easily reach the rebar surface, thereby inducing rebar depassivation, formation of corrosion products, and matrix structure damage. For cracked concrete, this pressure-driven penetration will further amplify the coupling effect between medium transmission and structure deterioration, making the durability problem more prominent.

At present, a lot of research has been conducted on chloride erosion, sulfate erosion, magnesium salt erosion, and concrete crack self-healing at home and abroad. Existing results generally believe that chloride ions can destroy the passive film on the rebar surface and induce electrochemical corrosion [16,17,18]. Sulfates and magnesium salts can react with cement hydration products to generate corrosion products such as AFt, thereby destroying the internal structure of concrete [19]. Under certain conditions, micro-cracks may also self-heal to a certain extent due to continued hydration and product deposition, thereby improving local impermeability [20,21,22]. However, most existing studies focus on the durability of ordinary concrete under non-pressure diffusion, dry–wet cycles, or capillary adsorption conditions. The understanding of the corrosion behavior of ecological concrete under the combined action of cracks and pressure-driven penetration in deep water environments is still insufficient, especially the lack of systematic research on the corrosion evolution law and micro-deterioration mechanism in the composite ion environment of chloride-sulfate-magnesium salt-bicarbonate.

Therefore, in this study, under the condition of constant water pressure, three corrosion conditions were set up: low-concentration chloride-sulfate, high-concentration chloride-magnesium sulfate, and high-concentration chloride-magnesium sulfate-carbonate composite. These conditions simulated the corrosive groundwater environment with low mineralization, the strongly corrosive groundwater environment with high mineralization, and the complex actual groundwater environment respectively. Then, a cyclic pressure-driven penetration test was carried out. By analyzing the chloride ion content distribution, penetration behavior evolution, changes in rebar half-cell potential, and macroscopic corrosion characteristics of concrete under different conditions, combined with micro-testing methods such as XRD and SEM, this study systematically reveals the corrosion behavior, crack healing-deterioration evolution characteristics, and micro-deterioration mechanism of ecological concrete in deep water environments, aiming to provide a theoretical basis for the durability design, service evaluation, and protection of ecological concrete structures in underground engineering and marine engineering.

## 2. Test Scheme

### 2.1. Raw Materials

#### 2.1.1. Cement

The cement used is Conch brand P·O 42.5 ordinary Portland cement produced by Foshan Conch Cement Co., Ltd. (Foshan, China). According to the requirements of Common Portland Cement (GB 175-2023) [23], the setting time, compressive strength, flexural strength of cement mortar, and other related indicators of cement were tested. The physical and mechanical properties of cement are shown in Table 1. The test results show that all performance indicators meet the requirements of ordinary Portland cement in the national standard (GB 175-2023).

#### 2.1.2. Fly Ash

Fly ash is provided by Shishi Xiezhong Building Materials Co., Ltd. (Foshan, China). According to the relevant provisions of Fly Ash Used for Cement and Concrete (GB/T 1596-2017) [24], the moisture content, fineness, water demand ratio, activity index, and other indicators of fly ash were tested. The test results of its physical properties are shown in Table 2. The test results show that all performance indicators of the fly ash meet the technical requirements of Class I fly ash in the national standard (GB/T 1596-2017), and can be used for concrete preparation in this test.

#### 2.1.3. Slag

The slag powder was Grade S95 slag powder produced by Tangshan Iron and Steel Plant., Ltd. (Tangshan, China). According to the requirements of Ground Granulated Blast Furnace Slag Powder Used for Cement and Concrete (GB/T 18046-2017) [25], the relevant indicators of the slag powder were tested, and its properties are shown in Table 3. For the performance requirements of Grade S95 slag powder specified in the standard, the 7-day activity index of the slag powder was slightly lower, while all other index met the requirements of Grade S95.

#### 2.1.4. Fine Aggregate

The fine aggregate was medium-coarse river sand. Its physical properties are shown in Table 4. The sand meets the requirements specified in the national standard Sand for Construction (GB/T 14684-2022) [26], and belongs to medium sand in Zone II of gradation, which can provide good workability and mechanical properties for concrete, meeting the needs of most construction projects.

#### 2.1.5. Coarse Aggregate

The coarse aggregate was artificial sandstone crushed stone with particle sizes being 5–31.5 mm. The apparent density, water absorption, needle and flake particle content, and other indicators of the crushed stone are shown in Table 5. The performance indicators of the coarse aggregate all meet the requirements for Class I coarse aggregate specified in the national standard Pebble and Crushed Stone for Construction (GB/T 14685-2022) [27], which is suitable for projects with high requirements for the strength and durability of concrete.

#### 2.1.6. Admixtures

Naphthalene-based water reducer (FDN-1), and the air-entraining agent was DH-9. The performance indicators are shown in Table 6.

### 2.2. Mix Proportion Design

Two groups of mix proportions with water–binder ratios of 0.33 and 0.38 were adopted. The calculation of the water–binder ratio complies with the requirements of Technical Code for Application of Fly Ash Concrete (GB/T 50146-2014) [28], as shown in Table 7.

### 2.3. Curing Duration and Crack Setting

The prepared concrete specimens were subjected to standard curing, which involves placing the specimens in an environment with a temperature of 20 ± 2 °C and a relative humidity of not less than 95%.

**Figure 1:**
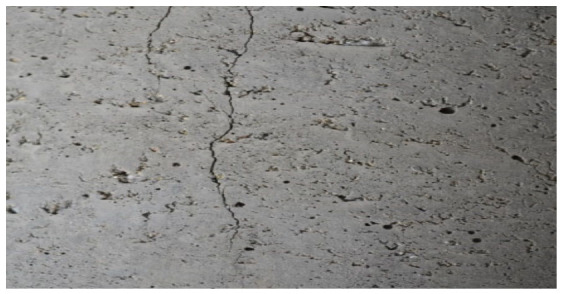
Surface pre-existing crack diagram of impermeability test specimen.

After the specimens were formed and cured under standard conditions for 7 days, specimens were split along the direction perpendicular to the reinforcement using a compressive strength testing machine to create cracked concrete. During splitting loading, careful observation was required; loading was stopped as soon as micro-cracks appeared on the surface to prevent excessively large surface cracks that would not simulate the experimental effect of micro-cracks. Splitting effect as shown in Figure 1. After splitting, the specimens continued standard curing until 28 days of age. A micrometer was used to measure the surface, ensuring that the maximum reserved splitting crack width was within 0.2 mm. Specimens exceeding this crack width were discarded.

### 2.4. Corrosion Environment Setting

To simulate the corrosion effect that concrete may suffer in deep water environments under different hydrochemical conditions, three types of corrosion solutions were prepared in this study based on the actual on-site situation after referring to relevant literature and materials, and their composition and content are shown in Table 8. The low-concentration chloride-sulfate solution was composed of NaCl and Na_2_SO_4_, with the contents of NaCl and Na_2_SO_4_ being 11.53 g/L and 4.43 g/L, respectively, which was mainly used to simulate the groundwater environment with low salinity and Cl^−^ and SO_4_^2−^ as the main corrosive ions. The high-concentration chloride-magnesium sulfate solution increased the chloride salt concentration on this basis and introduced MgCl_2_·6H_2_O, with the contents of NaCl, Na_2_SO_4_, and MgCl_2_·6H_2_O being 17.40 g/L, 4.43 g/L, and 12.68 g/L, respectively. It was mainly used to simulate the strongly corrosive groundwater or salt water seepage environment with high salinity, rich in Cl^−^, SO_4_^2−^, and Mg^2+^. The high-concentration chloride-magnesium sulfate-carbonate composite solution further added NaHCO_3_, with the contents of NaCl, Na_2_SO_4_, MgCl_2_·6H_2_O, and NaHCO_3_ being 9.09 g/L, 2.21 g/L, 4.22 g/L, and 0.19 g/L, respectively. It was used to simulate the composite ion-type groundwater environment containing Cl^−^, SO_4_^2−^, Mg^2+^, and HCO_3_^−^. By setting the above three corrosive media, the corrosion behavior and deterioration characteristics of cracked concrete in different hydrochemical corrosion environments can be compared and analyzed.

### 2.5. Test Methods

#### 2.5.1. Chloride Ion Content Test

In the test, the concrete specimens were sampled in layers along the depth direction at different ages (90 days, 180 days, 365 days), with the sampling depths being 5 mm, 15 mm, 25 mm, 35 mm, and 45 mm, respectively. After crushing and sieving the taken concrete samples, the water-soluble method was used to extract chloride ions, and the silver nitrate titration method was used to determine the content of water-soluble chloride ions, so as to analyze the migration and distribution law of chloride ions in concrete [29,30,31]. In this experiment, three samples were tested repeatedly, and the final results were averaged to ensure the accuracy of the measurements.

#### 2.5.2. Pressure-Driven Penetration Test

The pressure-driven penetration test was carried out with a concrete impermeability tester. According to Test code for hydraulic concrete (SL352-2020) [32], the impermeability test was used to analyze the concrete anti-permeability performance. To ensure that water seeps through at an appropriate measurable time under different test conditions, 0.3 MPa water pressure was applied to drive the corrosion solution to penetrate into the concrete interior through cracks and pores under external pressure.

**Figure 2:**
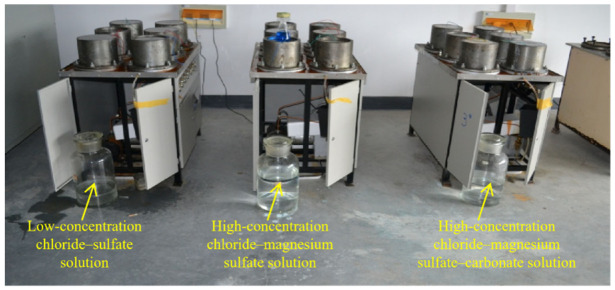
Cyclic pressure-driven penetration test device under different corrosion solution conditions.

To simulate the effect of different corrosion environments on the durability of cracked concrete, this study set three corrosive media: low-concentration chloride-sulfate solution, high-concentration chloride-magnesium sulfate solution, and high-concentration chloride-magnesium sulfate-carbonate composite solution, and used three impermeability testers to carry out cyclic pressure-driven penetration tests. The test device is shown in Figure 2. During the test, the surface of the specimens was regularly observed for leakage, and the penetration conditions at different ages (1month, 3 months, 6 months, 9 months, 12 months) were recorded to evaluate the penetration evolution characteristics and corrosion evolution law of cracked concrete in different corrosion environments. In this experiment, six samples were tested repeatedly, and the final results were averaged to ensure the accuracy of the measurements.

#### 2.5.3. Rebar Half-Cell Potential Test

The rebar corrosion state was monitored by the half-cell potential method. During the test, a test system composed of a copper/copper sulfate reference electrode and a high-impedance potentiometer was used to measure the electrode potential of the embedded rebar in the specimens. The rebar half-cell potential was measured every 6 days from day 22 to day 136, and the corrosion evolution degree of the rebar was evaluated by analyzing the change trend of the potential. In this experiment, two samples were tested repeatedly, and the final results were averaged to ensure the accuracy of the measurements.

#### 2.5.4. XRD Test

First, from the test group with water–binder ratio of 0.38, the reference specimen without pre-cracks, the specimen subjected to penetration test in the low-concentration corrosion solution, the specimen subjected to penetration test in the high-concentration corrosion solution, and the specimen subjected to penetration test in the composite carbonate corrosion solution were selected. To analyze the changes of concrete hydration products under the corrosion environment, cement mortar samples were taken from the specimens for the XRD test. The test adopted an X-ray diffractometer, with Cu Kα radiation as the radiation source, a wavelength λ = 0.15406 nm, a working voltage of 40 kV, and a working current of 40 mA. The samples were scanned at room temperature, with a scanning range of 5–70° (2θ), a step size of 0.02°, and a scanning rate of 5°/min. According to the changes in the position and relative intensity of diffraction peaks, the composition characteristics of hydration products and corrosion products in the samples under different corrosion conditions were analyzed.

#### 2.5.5. SEM Test

After the test, the specimens were split, and cement paste samples were taken for SEM observation to analyze the micro-morphology of cement paste and the characteristics of corrosion products. Before the test, the samples were dried and gold-sputtered on the surface to improve conductivity. The SEM test adopted the secondary electron imaging mode, with a working voltage of 15 kV, a working distance of about 10 mm, and an observation magnification of 500–5000 times. At the same time, energy dispersive spectroscopy (EDS) analysis was carried out on some split areas to determine the elemental composition of the main hydration products and corrosion products inside the specimens.

## 3. Corrosion Behavior of Cracked Concrete Under Pressure-Driven Penetration

### 3.1. Comparison Between Pressure-Driven Penetration and Capillary Penetration

To compare the migration characteristics of chloride ions in cracked concrete under pressure-driven penetration and capillary penetration conditions, the content of water-soluble chloride ions at different depths of specimens at different ages was determined; the data represent the average content measured from three specimens in each group, and the results are shown in Table 9.

**Figure 3:**
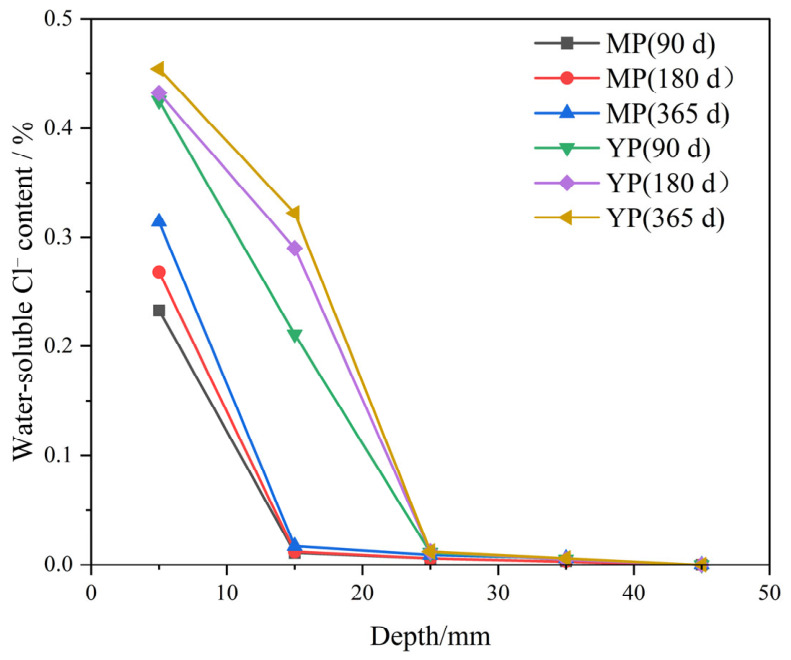
Depth distribution of water-soluble chloride content under different penetration conditions.

It can be seen from Figure 3 that under both penetration conditions, the chloride ion content in concrete decreased rapidly with the increase of depth, indicating that chloride ions were mainly concentrated in the surface layer of concrete. When the depth reached 25 mm, the chloride ion content in the specimens at all ages decreased significantly, and the chloride ion content in the area below 30 mm was close to zero, indicating that the diffusion capacity of chloride ions in the concrete interior was significantly limited.

In contrast, under the same age conditions, the chloride ion content of the pressure-driven penetration specimens (YP) was significantly higher than that of the capillary penetration specimens (MP). Taking the 90-day specimens as an example, at the depth of 5 mm, the chloride ion content of the YP specimen reached 0.425%, while that of the MP specimen was only 0.233%. At the depth of 15 mm, the chloride ion content of the YP specimen was 0.211%, which was significantly higher than 0.011% of the MP specimen. This result indicates that under the action of external water pressure, the corrosion solution can more easily enter the concrete interior along cracks and pores, thereby significantly improving the migration rate and penetration depth of chloride ions.

From the perspective of age change, whether under capillary penetration or pressure-driven penetration conditions, with the increase of age from 90 d to 365 d, the chloride ion content in the concrete surface layer increased to some extent. For example, under pressure-driven penetration conditions, the chloride ion content at the depth of 5 mm increased from 0.425% at 90 d to 0.454% at 365 d, and that at the depth of 15 mm increased from 0.211% to 0.322%. This indicates that with the extension of erosion time, chloride ions continuously migrate into the concrete interior and gradually accumulate.

Overall, the migration capacity of chloride ions under pressure-driven penetration conditions was significantly stronger than that under capillary penetration conditions. This not only led to a significant increase in the chloride ion concentration in the concrete surface layer, but also enabled chloride ions to enter the concrete interior in a shorter time, thereby accelerating the rebar corrosion process [33]. This indicates that in practical engineering, when concrete structures are in groundwater pressure or pressure-driven penetration environments, using only traditional diffusion or capillary adsorption models to evaluate chloride ion erosion may underestimate the corrosion risk of the structure. It is noteworthy that there are significant differences in the mechanisms between capillary penetration and pressure-driven permeation. Capillary transport is primarily driven by capillary suction and concentration gradients, leading to the slow and passive infiltration of solutions. This process largely depends on pore structure and surface tension within the concrete matrix. Pressure-driven transport is a process primarily driven by external hydraulic pressure, which actively forces solutions through cracks and pores at higher rates and greater depths, significantly accelerating the migration process. This distinction is crucial for interpreting experimental results and evaluating the durability of concrete under different environmental conditions, as the two mechanisms represent distinctly different environments when compared.

### 3.2. Corrosion Evolution Law of Cracked Concrete Under Different Corrosion Solution Conditions

#### 3.2.1. Comparison of Leakage and Crack Change Characteristics

Through a one-year cyclic pressure-driven penetration test, the penetration conditions of each specimen are shown in Table 10. The results show that the uncracked specimens never showed penetration phenomenon under the action of 0.3 MPa cyclic pressure, indicating that no through cracks were formed inside the specimens, and the structure remained intact. While in the high-concentration chloride-magnesium sulfate solution and high-concentration chloride-magnesium sulfate-carbonate composite solution environments, the pre-cracked specimens were in a continuous penetration state throughout the test period, indicating that the cracks remained through, and corrosive media could continuously enter the concrete interior.

In contrast, the specimens tested in the low-concentration chloride-sulfate solution only showed penetration phenomenon in the early stage of the test, and the penetration gradually stopped before the age of about 3 months, and no penetration occurred again until the end of the test. This phenomenon is consistent with the aforementioned “U-shaped” change law of the rebar electrode potential curve. The reason may be that with the continuous progress of concrete hydration reaction, the generated hydration products gradually fill and block the crack channels, thereby blocking the further penetration of the corrosion solution, and making the rebar re-enter the passive state [34].

The test results show that when the crack width is less than 0.2 mm, the cracks have a certain self-healing capacity in the low-concentration chloride-sulfate environment. While in the high-concentration chloride-magnesium sulfate solution and high-concentration chloride-magnesium sulfate-carbonate composite solution environments, the corrosion effect continues to increase, the cracks are difficult to close, and the corrosion damage of reinforced concrete is intensified, so it is necessary to timely seal and repair the cracks.

#### 3.2.2. Comparison of Evolution Law of Half-Cell Potential

**Figure 4:**
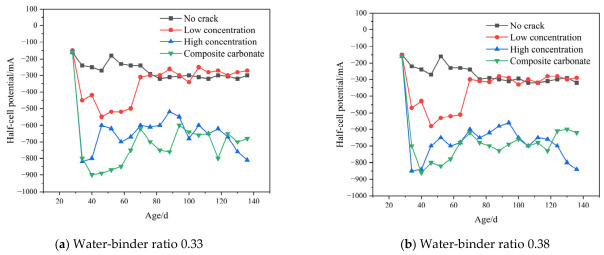
Rebar electrode potential of concrete specimens eroded by different corrosion solutions at different ages.

To study the corrosion evolution law of reinforced concrete under different corrosion environment conditions, the half-cell potential of the rebar embedded in the concrete specimens was regularly detected, once every 6 days, until 136 d. The detection results of the specimens with a water–binder ratio of 0.33 and the specimens with a water–binder ratio of 0.38 and air entrainment are shown in Figure 4. In the figure, the uncracked group is the control specimen without pre-cracks in the early stage of the test. The low-concentration group is the specimen with low-concentration chloride-sulfate solution as the corrosive medium. The high-concentration group is the specimen in the high-concentration chloride-magnesium sulfate solution. The composite carbonate group is the specimen in the high-concentration chloride-magnesium sulfate-carbonate composite solution.

It can be seen from the test results that without pre-cracks, the change range of the rebar half-cell potential was small. The potential of the specimen with a water–binder ratio of 0.33 was mainly distributed between −160 and −320 mV during the whole test period, and that of the specimen with a water–binder ratio of 0.38 was mainly distributed between −150 and −320 mV, which remained in a relatively stable range on the whole, indicating that corrosive media were difficult to enter the concrete interior, and the rebar basically did not undergo obvious corrosion.

In the low-concentration chloride-sulfate solution environment, the absolute value of the rebar half-cell potential increased rapidly in the early stage of the test, and then gradually decreased and tended to be stable. For the specimen with a water–binder ratio of 0.33, the potential decreased rapidly from −150 mV at 28 d to −450 mV at 34 d, and reached −550 mV at 46 d, then gradually rebounded, and basically stabilized between −250 and −340 mV during 70–136 d. For the specimen with a water–binder ratio of 0.38, the potential decreased from −150 mV at 28 d to −470 mV at 34 d, reached −580 mV at 46 d, then gradually rebounded, and stabilized around −280–−330 mV during 70–136 d. Combined with the penetration test phenomenon, it can be seen that in the early stage of the test, the corrosion solution could enter the concrete interior along the pre-cracks, making the rebar in an active corrosion state. With the progress of the test, the cracks were gradually filled and closed by hydration products and corrosion reaction products, the penetration of the corrosion solution gradually stopped, the rebar was separated from the corrosive media again, and the potential tended to be stable accordingly.

In contrast, in the high-concentration chloride-magnesium sulfate solution and high-concentration chloride-magnesium sulfate-carbonate composite solution environments, the absolute value of the rebar half-cell potential increased rapidly in the early stage of the test, and continued to fluctuate within a high corrosion potential range, indicating that the rebar was in a continuous corrosion state. Taking the specimen with a water–binder ratio of 0.33 as an example, the potential of the high-concentration group reached −820 mV and −800 mV at 34 d and 40 d, respectively, and although there were some fluctuations afterwards, it basically remained between −520 and −810 mV during 70–136 d. The composite carbonate group was −800 mV and −900 mV at 34 d and 40 d, respectively, and still remained between −600 and −800 mV in the later stage. For the specimen with a water–binder ratio of 0.38, the high-concentration group reached −850 mV and −840 mV at 34 d and 40 d, respectively, and was still −840 mV at 136 d. The composite carbonate group reached −860 mV at 40 d, and basically remained between −600 and −730 mV during 70~136 d. This indicates that in the high-concentration and composite corrosion environments, the cracks always maintained strong connectivity, and corrosive media could continuously enter the concrete interior and accelerate rebar corrosion. Overall, the potential change laws of the two groups of specimens with different water–binder ratios were basically consistent. The uncracked group was always relatively stable, the low-concentration group showed the characteristics of first activation and then stabilization, while the high-concentration group and composite carbonate group always maintained a high corrosion potential level, indicating that their corrosion evolution was more continuous and significant.

#### 3.2.3. Analysis of Differences in Corrosion Evolution Degree

**Figure 5:**
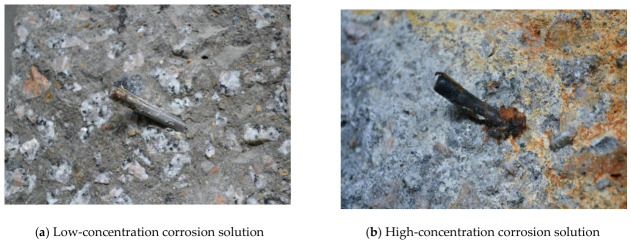
Rebar corrosion in corrosion solutions with different concentrations.

To further analyze the corrosion evolution degree of reinforced concrete under different corrosion environments, the specimens were split after the test, and the corrosion condition of the rebar surface was observed; the results are shown in Figure 5. It can be seen from the figure that in the low-concentration corrosion solution environment, the rebar surface basically maintained the original shape, only local slight oxidation traces appeared, and the overall corrosion degree was light. This indicates that in this corrosion environment, the erosion effect of corrosive media on the rebar was weak.

In contrast, in the high-concentration corrosion solution environment, obvious corrosion products appeared on the rebar surface, and obvious corrosion traces could also be observed on the surrounding concrete surface, and the rebar corrosion degree was significantly intensified. This indicates that under the action of high-concentration corrosive media, corrosion ions can more easily enter the concrete interior through cracks, and continuously react with the rebar, thereby accelerating the rebar corrosion process.

Combined with the aforementioned change results of rebar half-cell potential, it can be seen that the change of rebar potential in the low-concentration corrosion environment gradually tended to be stable, indicating that the corrosion evolution was inhibited to a certain extent. Meanwhile, in the high-concentration corrosion environment, the rebar potential was always at a high corrosion level, indicating that the rebar was in a continuous corrosion state. The macroscopic corrosion phenomenon was in good consistency with the potential detection results.

## 4. Healing-Deterioration Characteristics of Cracked Reinforced Concrete Under Corrosion-Penetration Coupling Action

### 4.1. Characteristics of Corrosion Evolution Under Pressure-Driven Penetration Conditions

Through comprehensive analysis of chloride ion distribution, penetration phenomenon, changes in rebar half-cell potential, and macroscopic corrosion conditions, it can be seen that the corrosion evolution of cracked concrete under pressure-driven penetration conditions has obvious stage-specific characteristics and environment-dependent characteristics. Compared with non-pressure capillary penetration, the external water pressure significantly enhanced the migration capacity of corrosive media in concrete, making the corrosion solution easier to enter the concrete interior along cracks and pores, thereby accelerating the transmission of chloride ions to the rebar position.

In the low-concentration chloride-sulfate corrosion environment, the corrosion solution could penetrate into the concrete interior through cracks in the early stage of the test, making the rebar half-cell potential develop rapidly in the negative direction, which shows a certain corrosion trend. But with the progress of the test, the continuous hydration inside the concrete and the expansive products generated by the corrosion reaction gradually filled the crack channels, making the cracks self-heal to a certain extent, the penetration of the corrosion solution gradually weakened or even stopped, the rebar potential tended to be stable, and the corrosion evolution was inhibited to a certain extent.

In contrast, in the high-concentration chloride-magnesium sulfate solution and high-concentration chloride-magnesium sulfate-carbonate composite solution environments, the concentration of corrosive media was high, and the chemical erosion effect was more significant. Under the action of pressure-driven penetration, the corrosion solution could continuously enter the concrete interior through cracks, making the rebar in a corrosion activation state for a long time, and the half-cell potential remained at a high corrosion level. At the same time, the combined action of sulfate, magnesium ions, and carbonate ions would further destroy the structure of cement hydration products, gradually deteriorate the internal structure of concrete, and make it difficult for cracks to be effectively closed by secondary hydration products, thereby leading to the continuous evolution of rebar corrosion.

### 4.2. Analysis of the Reasons for the Difference in Healing Phenomenon Under Different Corrosion Solution Conditions

#### 4.2.1. XRD Analysis

**Figure 6:**
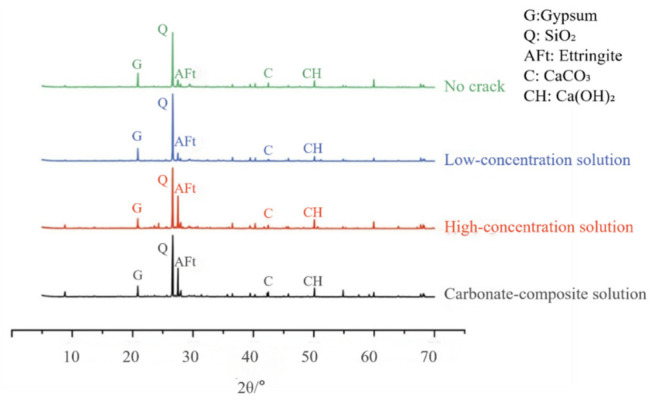
Comparison of XRD patterns of concrete specimens in different corrosive media.

To clearly explore the deterioration mechanism of concrete in corrosive media, the results of the XRD test were analyzed. The XRD comparison spectra are shown in Figure 6.

It can be seen from Figure 6 that the types of diffraction peaks of each specimen under different corrosion environments were generally similar, indicating that their main phase composition was basically the same. Characteristic peaks such as SiO_2_, AFt, CaCO_3_, and Ca(OH)_2_ could be detected in all samples. Among them, the SiO_2_ peak mainly came from the quartz component of the aggregate in the mortar; the Ca(OH)_2_ peak indicated that a certain amount of cement hydration products remained in the specimen; the CaCO_3_ peak indicated that there was a certain degree of carbonate formation or deposition inside the specimen. In contrast, the obvious difference between the groups was mainly reflected in the change of the intensity of the AFt characteristic peak. The AFt peaks in the uncracked group and the low-concentration solution group were relatively weak, while the AFt peaks in the high-concentration solution group and the carbonate composite solution group were more obvious, indicating that under the combined action of high-concentration corrosive media and pressure-driven penetration, sulfate was more likely to invade the concrete interior and react with hydration products to generate more expansive corrosion products such as AFt. The spectrum of the low-concentration group was close to that of the uncracked group, indicating that the internal corrosion reaction degree was relatively limited, which was consistent with the phenomenon that the penetration stopped in the later stage and the cracks self-healed to a certain extent. In addition to the obvious AFt peak, the carbonate composite solution group also showed a certain CaCO_3_ characteristic peak, indicating that while the sulfate erosion continued to develop in this group of specimens, it was accompanied by the formation of a certain amount of carbonate, reflecting that the internal deterioration mechanism of concrete under the composite corrosion environment was more complex.

#### 4.2.2. SEM Analysis of Specimens Under Low-Concentration Conditions

**Figure 7:**
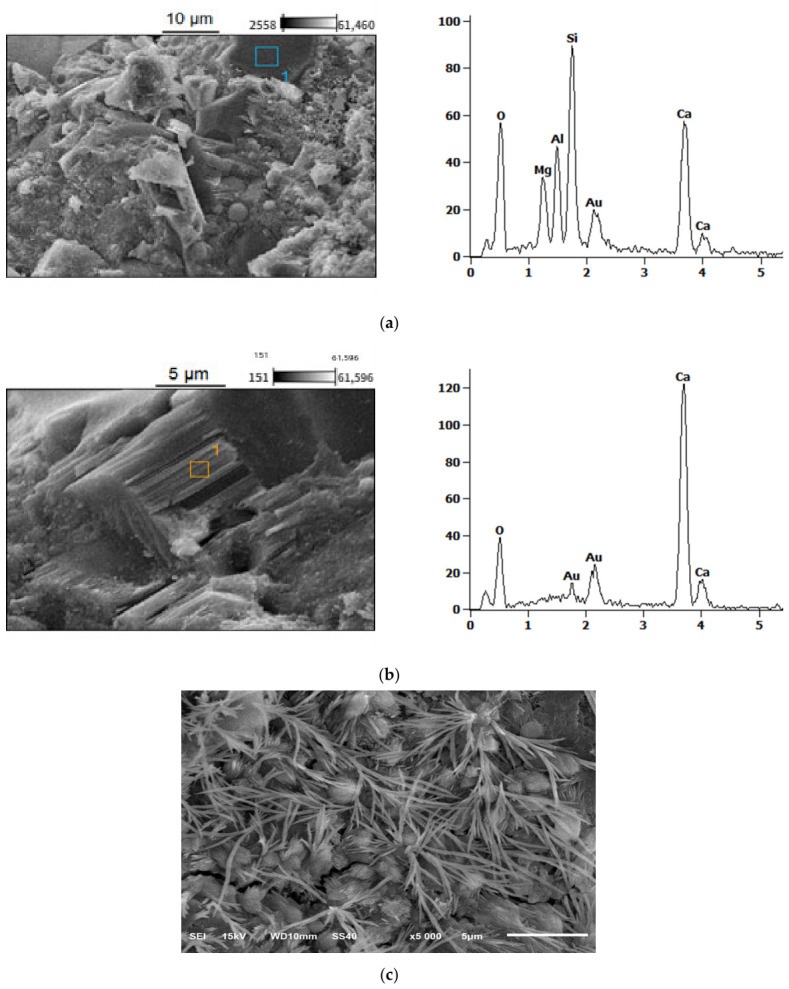

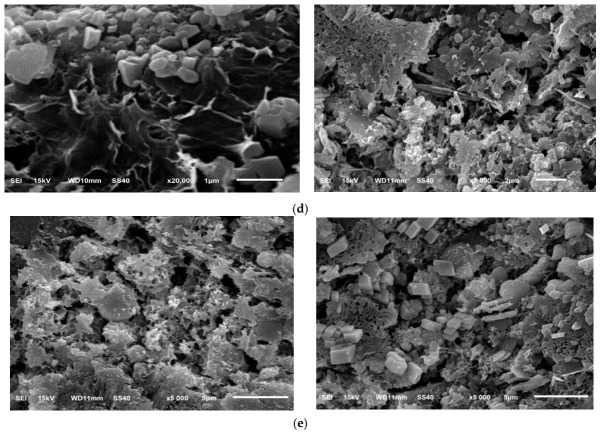
Hydration products of specimens in low-concentration chloride-sulfate solution. (**a**) C-S-H Products; (**b**) Calcium hydroxide; (**c**) AFt and expansive hydration products; (**d**) Secondary hydration products of slag; (**e**) Secondary hydration products of fly ash.

After the test, the specimens in the impermeability test device were unloaded and split, and cement paste samples were taken from the specimens for SEM observation, and combined with EDS energy spectrum to analyze the composition of hydration products in the hardened cement paste. The SEM image and partial EDS analysis results of the cement paste of the specimen under the low-concentration chloride-sulfate solution environment are shown in Figure 7. It can be seen from the figure that a large amount of calcium silicate hydrate (C-S-H) gel (marked position 1 in Figure 7a) and calcium aluminate hydrate gel were generated in the specimen, and there were also many calcium hydroxide crystals (marked position 1 in Figure 7b). In addition, the needle-like AFt formed under the action of sulfate ions interweaved with each other and presented a network structure, which together with the expansive hydration products played a role in filling and blocking the internal cracks of concrete. This microstructural feature was consistent with the macroscopic test results; that is, the cracks gradually self-healed during the test, and the corrosion solution was difficult to continue to penetrate through the cracks. Since the hydration products were not consumed in large quantities by the continuous corrosion reaction, the interior of the concrete could still maintain a high alkalinity, so that the passive film on the rebar surface was re-formed, thereby inhibiting further corrosion of the rebar. At the same time, the specimen subjected to the penetration test in the low-concentration chloride-sulfate corrosion environment no longer showed penetration phenomenon in the later stage, and the rebar half-cell potential gradually developed towards stability, indicating that the corrosive media were gradually separated from the rebar. Comprehensive analysis shows that under the combined action of continuous hydration reaction of concrete and corrosion reaction products, the crack channels are filled by hydration products and expansive crystals, realizing the self-healing of cracks.

The main reason for this phenomenon is as follows: In the initial stage of the experiment, the corrosive solution seeped into the concrete specimens through the pre-reserved cracks. The steel bars were in full contact with the corrosive solution. Chloride ions activated the passive film on the surface of the steel bars, triggering an electrochemical reaction. The electrode potential of the steel bars increased negatively, and the steel bars started to enter the corrosion stage. As the reaction progressed further, sulfates reacted with the cement gel to form expansive hydration products such as gypsum. In addition, a large amount of ettringite produced by the reaction could form a network structure, which combined with the secondary hydration products of slag and fly ash to block the pre-reserved cracks. At this time, the interior of the concrete was separated from the corrosive solution again, and the passive film on the steel bars was reformed.

#### 4.2.3. SEM Analysis of Specimens Under High-Concentration Conditions

**Figure 8:**
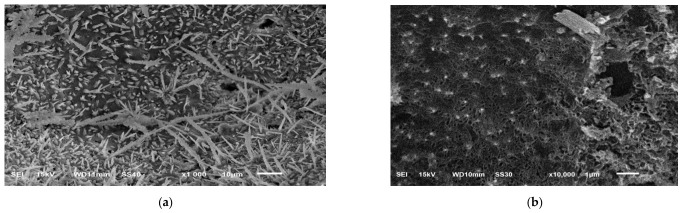
Hydration products of the specimen in the high-concentration chloride-magnesium sulfate solution. (**a**) Needle-like AFt structure; (**b**) Magnesium hydroxide (white spots).

As shown in Figure 8, in the high-concentration chloride-magnesium sulfate corrosion environment, a large number of needle-like AFt crystals can be observed in the hardened cement paste of the specimen, and there are also many white granular deposits. The AFt crystals present an obvious acicular or needle-like structure, and interweave with each other to form a relatively dense crystal network, indicating that under the combined action of sulfate ions and magnesium ions, a significant sulfate erosion reaction occurred inside the concrete. With the continuous progress of the corrosion reaction, the C-S-H gel in the cement hydration products was gradually destroyed, and new corrosion products were generated. The continuous generation and growth of a large number of AFt crystals inside the concrete will generate a large crystallization expansion pressure, thereby leading to gradual cracking of the internal structure of concrete and obvious deterioration.

Under the cyclic pressure-driven penetration conditions, the high-concentration chloride-magnesium sulfate solution can quickly penetrate through the specimen along the crack channels, and maintain a continuous penetration state throughout the test period. The rebar half-cell potential was always in a high corrosion potential range, indicating that the rebar was in a continuous corrosion state. After the test, the split observation of the specimen found that obvious corrosion phenomenon had appeared on the rebar surface. Compared with the low-concentration chloride-sulfate solution environment, the deterioration degree of the specimen in the high-concentration chloride-magnesium sulfate solution was significantly more serious. This was not only related to the higher chloride ion concentration, but also closely related to the participation of magnesium ions. Magnesium ions can reduce the internal alkalinity of concrete and destroy the rebar passive film, and at the same time continuously destroy the C-S-H gel structure, making it difficult for concrete to realize crack filling and self-healing through secondary hydration products, thereby leading to long-term cyclic penetration of the corrosion solution in the crack channels, and finally intensifying the deterioration of the concrete structure.

#### 4.2.4. SEM Analysis of Specimens Under High-Concentration Composite Carbonate Solution

**Figure 9:**
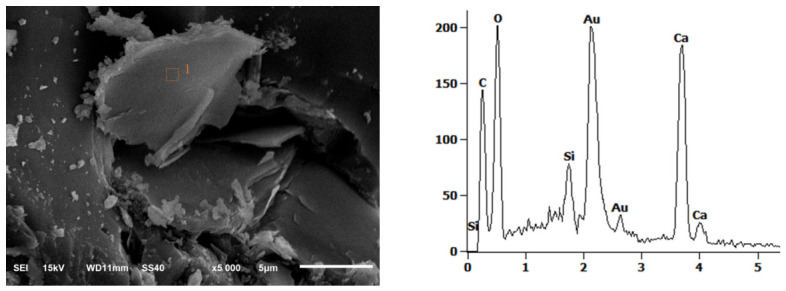
Hydration products of the specimen in the high-concentration chloride-magnesium sulfate-carbonate solution.

As shown at the location marked 1 in Figure 9, in the high-concentration chloride-magnesium sulfate-carbonate composite corrosion environment, an obvious flaky or plate-like crystal structure can be observed in the deep region of the split specimen. Combined with the characterization results from EDS energy spectrum analysis of the deep region of the specimen, it is known that this area primarily consists of elements such as Ca, C, and O, indicating that its main component is calcium carbonate crystals. This indicates that the cement hydration products in the deep region of the sample are also affected by the solution, leading to the formation of calcium carbonate. The specimen undergoes complex deterioration reactions with the aforementioned corrosive solution. The calcium hydroxide in the cement hydration products is largely consumed and gradually transformed into calcium carbonate precipitate. Since the generated calcium carbonate crystals lack effective cementing capacity, it is difficult to maintain the structural stability of the original cement-based materials, thereby making the internal structure of concrete gradually loose.

In the composite corrosion environment, chloride ions, sulfate ions, magnesium ions, and carbonate ions jointly participate in the corrosion reaction, making the concrete deterioration process more complex. On the one hand, the carbonate action will reduce the internal alkalinity of concrete, making the passive film on the rebar surface easier to be destroyed, thereby accelerating the corrosion effect of chloride ions on the rebar. On the other hand, the magnesium ions react with calcium hydroxide in concrete, causing a decalcification phenomenon, which in turn reduces the alkalinity inside the concrete. As the alkalinity decreases, the high-pH environment around the steel bars is damaged, which reduces the stability of the passive film on the steel bars that were originally in a passivated state. Eventually, the passive film is destroyed. The synergistic action of multiple corrosive media leads to continuous deterioration of the internal structure of concrete, and under the pressure-driven penetration conditions, the corrosion solution can continuously enter the concrete interior, thereby making the rebar in the corrosion environment for a long time, and finally leading to relatively serious corrosion damage of the specimen.

## 5. Conclusions

The main conclusions are as follows.

(1) Under both pressure-driven permeation and unpressurized capillary penetration, the Cl^−^ content in concrete decreased rapidly with increasing depth and was mainly concentrated in the surface layer. A marked reduction was observed beyond 25 mm, and the Cl^−^ content at depths of 35 mm and below was close to zero. At the same age, the Cl^−^ content in specimens subjected to pressure-driven permeation was significantly higher than that in capillary-penetration specimens. For example, at 90 d, the Cl^−^ content at 5 mm increased from 0.233% to 0.425%, and that at 15 mm increased from 0.011% to 0.211%; at 365 d, the value at 15 mm increased from 0.017% to 0.322%. These results indicate that external water pressure significantly enhanced the migration of corrosive media in cracked concrete.

(2) In the low-concentration chloride-sulfate environment, permeation was observed in cracked specimens at the initial stage. However, as hydration products and corrosion products gradually filled the cracks, a certain degree of self-healing was exhibited. Permeation almost ceased after about 3 months, the steel half-cell potential tended to stabilize, and corrosion development was restrained to some extent.

(3) In the high-concentration chloride-magnesium sulfate environment and the high-concentration chloride-magnesium sulfate-carbonate composite environment, the cracks remained continuously open, and the corrosive solution kept penetrating throughout the entire test period. The steel half-cell potential remained at a high corrosion level for a prolonged period, and obvious rusting was observed on the steel surface, indicating that the combined action of a high-concentration corrosive environment and pressure-driven permeation significantly aggravated steel corrosion.

(4) The XRD and SEM results showed that, in the low-concentration environment, products such as C-S-H gel, Ca(OH)_2_, and AFt filled the cracks and made the structure denser. In contrast, under high-concentration and composite environments, large amounts of AFt, Mg(OH)_2_, and CaCO_3_ were formed. The combined action of sulfate, magnesium ions, and carbonate ions damaged the C-S-H gel and reduced the internal alkalinity, leading to continuous deterioration of the concrete microstructure and a significant decline in structural durability. However, given that these microstructural analyses are primarily qualitative, further quantitative research is still needed to fully elucidate the detailed microscopic degradation mechanisms.

## Figures and Tables

**Table 1:** Physical and mechanical properties of cement.

Raw Material	Water Requirement of Normal Consistency /%	Compressive Strength /MPa		Flexural Strength /MPa		Setting Time /min		Soundness
		3 d	28 d	3 d	28 d	Initial Setting	Final Setting	
Cement	26.7	29.2	54.8	7.0	11.8	109	224	Qualified
GB 175-2023	—	≥17.0	≥42.5	≥3.5	≥6.5	≥45	≤600	Qualified

**Table 2:** Physical properties of fly ash.

Physical Property	Moisture Content (%)	Density(g·cm^−3^)	Fineness (%)	Water Demand Ratio (%)	Activity Index (%)
Materials Test results	0.06	2.31	6.2	95	82.6
Standard	≤1.0	—	≤12.0	≤95	≥70.0

**Table 3:** Physical properties of slag powder.

Physical Property	Moisture Content (%)	Density (g/cm^3^)	Specific Surface Area (m^2^/kg)	Fluidity Ratio (%)	Activity Index/%	
					7 d	28 d
Slag powder	0.07	2.86	436	98.2	72.2	96.1
S95 Standard	≤1.0	≥2.8	≥400	≥95	≥75	≥95
S75 Standard	≤1.0	≥2.8	≥300	≥95	≥55	≥75

**Table 4:** Physical properties of fine aggregate.

Fine Aggregate	Fineness Modulus	Apparent Density /(g/cm^3^)	Saturated Surface Dry Density /(g/cm^3^)	Saturated Surface Dry Water Absorption/%	Clay Content /%
Sand	2.80	2.62	2.60	0.73	2.52
GB/T 14684-2022	—	≥2.50	—	—	≤3.0 (II)

**Table 5:** Main performance indicators of coarse aggregate.

Coarse Aggregate	Apparent Density /(g/cm^3^)	Water Absorption /%	Needle and Flake Particle Content /%	Clay Content /%	Clod Content /%	Crushing Index /%
Artificial crushed stone	2.68	0.51	1.23	0.3	0	9.0
GB/T 14685-2022	≥2.60	≤1.0 (I)	≤5 (I)	≤0.5 (I)	0 (I)	≤10 (I)

**Table 6:** Performance test results of admixtures.

Test Item		Water Reducer	Air-Entraining Agent
Solid content/%		35	12
Dosage/%		0.7	0.06
Water reduction rate/%		20.0	6.8
Bleeding rate ratio/%		46	31
Air content/%		1.9	5.5
Setting time difference /min	Initial setting	+25	−30
	Final setting	+15	−40
Compressive strength ratio/%	1 d	180	—
	3 d	170	97
	7 d	168	97
	28 d	157	92
Shrinkage ratio/%		102	119

**Table 7:** Test mix proportions/kg/m^3^.

Water–Binder Ratio	Water	Cement	Fly Ash	Slag Powder	Sand	Small Stone	Medium Stone	Water Reducer	Air-Entraining Agent
0.33	156	189	95	189	658	438	657	3.8	0
0.38	150	158	79	158	715	419	629	2.8	0.43

**Table 8:** Composition and content of corrosion solutions.

Solution Type	NaCl (g/L)	Na_2_SO_4_ (g/L)	MgCl_2_·6H_2_O (g/L)	NaHCO_3_ (g/L)	Corresponding Water Environment
Low-concentration chloride-sulfate solution	11.53	4.43	-	-	Lowly mineralized groundwater environment
High-concentration chloride-magnesium sulfate solution	17.42	4.43	12.68	-	Highly mineralized strongly corrosive water environment
High-concentration chloride-magnesium sulfate-bicarbonate composite solution	9.09	2.21	4.22	0.19	Composite groundwater environment

**Table 9:** Water-soluble chloride content in concrete mortar under pressure-driven and capillary penetration.

Depth (mm)	MP (Non-Pressure Capillary Permeation)			YP (Pressure Permeation)		
	90 d	180 d	365 d	90 d	180 d	365 d
5	0.233	0.268	0.314	0.425	0.432	0.454
15	0.011	0.012	0.017	0.211	0.29	0.322
25	0.006	0.006	0.009	0.011	0.012	0.012
35	0.003	0.003	0.006	0.005	0.005	0.006
45	0.000	0.000	0.000	0.000	0.000	0.000

**Table 10:** Comparison of penetration phenomenon of specimens under different corrosion solution conditions.

Test Condition	Age				
	1 Month	3 Months	6 Months	9 Months	12 Months
No crack	No permeation	No permeation	No permeation	No permeation	No permeation
Low concentration	Permeation	No permeation	No permeation	No permeation	No permeation
High concentration	Permeation	Permeation	Permeation	Permeation	Permeation
Composite carbonation	Permeation	Permeation	Permeation	Permeation	Permeation

## Data Availability

The original contributions presented in this study are included in the article. Further inquiries can be directed to the corresponding author.
